# The economic benefits of increased sugar-free chewing gum in China: a budget impact analysis

**DOI:** 10.1186/s12903-021-01786-8

**Published:** 2021-09-07

**Authors:** Shuo Du, Chunzi Zhang, Wenhui Wang, Jian Liu, Chao Yuan, Yizhen Yu, Qing Chang, Shanshan Zhang, Yan Si

**Affiliations:** 1 Department of Preventive Dentistry, National Engineering Laboratory for Digital and Material Technology of Stomatology, Beijing Key Laboratory of Digital Stomatology, National Clinical Research Center for Oral Diseases, Research Center of Engineering and Technology for Digital Dentistry of Ministry of Health, Peking University School and Hospital of Stomatology, Beijing, 100081 China; 2 Department of Second Clinical Division, National Engineering Laboratory for Digital and Material Technology of Stomatology, Beijing Key Laboratory of Digital Stomatology, National Clinical Research Center for Oral Diseases, Research Center of Engineering and Technology for Digital Dentistry of Ministry of Health, Peking University School and Hospital of Stomatology, Beijing, China

**Keywords:** China, Sugar-free gum (SFG), Caries prevention, Budget impact analysis

## Abstract

**Background:**

To analyze the potential cost savings in dental care associated with increased sugar-free gum (SFG) use among Chinese teenagers and adults.

**Methods:**

The amount of SFG chewed per year and decayed, missing and filled teeth (DMFT) was collected from a cross-sectional survey to create a dose–response curve assumption. A cost analysis of dental restoration costs was carried out. A budget impact analysis was performed to model the decrease in DMFT and the subsequent cost savings for dental care. Three different scenarios for the increase in the number of SFG were calculated.

**Results:**

The average cost savings per person in the Chinese population due to increasing SFG use ranged from 45.95 RMB (6.94 USD) per year to 67.41 RMB (10.19 USD) per year. It was estimated that 21.51–31.55 billion RMB (3.25–4.77 billion USD) could be saved annually if all SFG chewers among Chinese teenagers and adults chewed SFG regularly.

**Conclusion:**

This study suggests that dental care costs could be significantly reduced if SFG use increased in the Chinese population.

**Supplementary Information:**

The online version contains supplementary material available at 10.1186/s12903-021-01786-8.

## Background

Oral conditions affect 3.9 billion people worldwide, and untreated caries in permanent teeth are the most prevalent condition accounted for by the global burden of disease studies [1, 2]The treatment of oral diseases is the 4th greatest expense in most industrial countries [3]. In 2010, the global economic impact of dental diseases amounted to US$442 billion, of which US$298 billion was the direct treatment cost, corresponding to an average of 4.6% of the global health expenditure [4]. In China, oral diseases have also caused a great economic burden [5, 6]. From 2005 to 2015, the expenditure on oral diseases in China increased by more than 10% [7].

Unlike the public approach of using fluoride to prevent dental caries at the national or regional level [8], chewing gum, as a mechanical aid to remove oral biofilm, is a preventive measure to enhance dental health at the individual level. The European Food Safety Authority (EFSA) has officially included chewing gum in its recommendations for a balanced diet [9]. There is evidence of a causal relationship between sugar-free gum (SFG) consumption and reduced tooth demineralization and between SFG consumption and reduced incidence of dental caries [10, 11]. Dental demineralization may increase the risk of caries [12]. Chewing gum stimulates the secretion of saliva, and as the flow rate of saliva increases, so does the concentration of calcium, phosphate and bicarbonate in the saliva, which is conducive to the remineralization of dental crystals [13]. In addition, chewing SFG can also improve the removal of food debris from the mouth, increase the pH of dental plaque, and reduce dry mouth and gingival inflammation [14, 15].

To solve the contradiction between the scarcity of dental healthcare resources and unlimited demand, it is particularly important to evaluate the health economics of new and existing health care interventions [16]. Through a comparison of different dental intervention programs, limited resources can be maximized, and a waste of resources can be avoided [17].

However, globally, there are few studies on the cost–benefit economic analysis of SFG in preventing caries [18–20], and information on this issue is still lacking in China. The aim of this study was to estimate the savings in dental treatment costs resulting from increased SFG use among Chinese teenagers and adults.

## Methods

The study focused on the potential cost savings of caries treatment among all SFG consumers in China if more SFG were chewed. A cross-sectional survey was conducted to collect data on dental treatment costs, the frequency of chewing SFG and caries status in the last 12 months. On this basis, the relationship between the level of dental caries and annual SFG consumption was assumed. The total annual expenditure on the treatment of dental caries that could have been avoided by increasing SFG usage was estimated by evaluating increasing SFG use in various scenarios. Outcomes were assessed over a one-year time period.

### Survey subjects and contents

Considering that the oral health care consciousness and the use of SFG may vary in areas with different levels of economic development, we roughly divided into economically developed areas (eastern of China) and economically less developed areas (central and western of China) according to the regional economic development level. Then choose two representative provinces in each area, namely Beijing and Guangdong (economically developed area), Hubei and Xinjiang (economically underdeveloped area). Each province including one area of interest was selected in urban and rural areas, and then a certain number of areas of interest were selected that covered schools and communities. Cluster sampling was conducted on a class and community basis. A total of 860 teenagers (12–15 years) took part in the survey with their consents and legal guardians’ consents, and 490 adults (18 years and over) signed the consent forms before the participation. The study protocol was approved by Peking University Stomatological Hospital Biomedical Ethics Committee. (No. PKUSSIRB-201942018) The survey included information on dental treatment costs and SFG chewing frequency over the past year; this information was obtained through a questionnaire survey (Additional files 2, 3). All the subjects received the oral health examination by visual examination combined with probing under the artificial light using plane mouth mirrors and Community Periodontal Index (CPI) probe. The prevalence data of caries(cavitated dentine lesions) were collected by clinical examination according to methods and the standardized criteria of the WHO [21]. In each province, three trained licensed dentists who had been calibrated by the training of the 4th NOHS under WHO guidelines performed the examination. Kappa values were 0.80 ~ 0.96. The examiners were blinded to the results of questionnaires including the chewing condition of each subject.

### The relationship between DMFT and annual SFG consumption

**Fig. 1:**
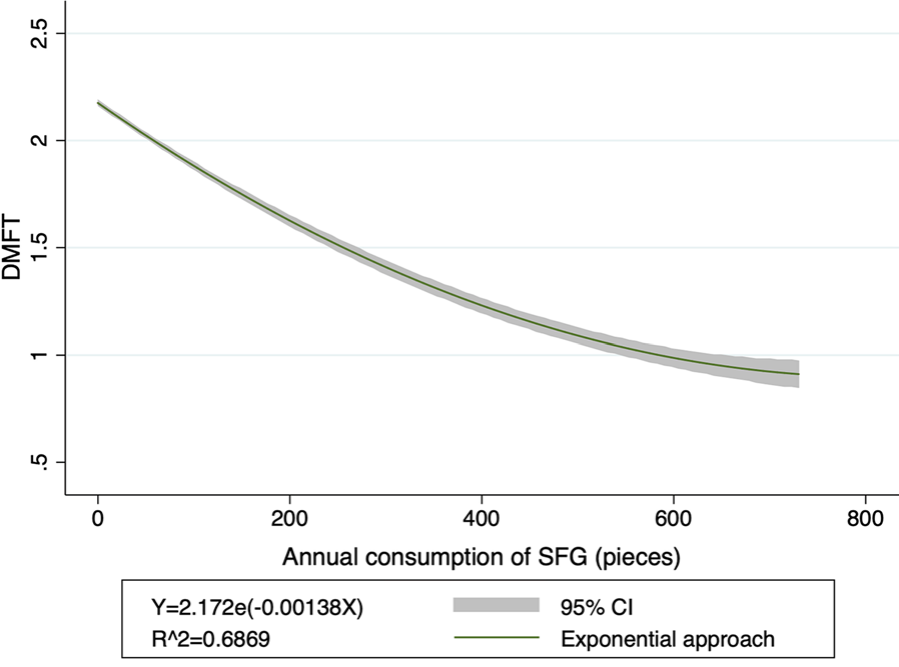
Relationship between annual SFG consumption and DMFT. Abbreviations: SFG, Sugar-Free Gum; DMFT, decayed, missing and filled teeth in permanent dentition; CI, confidence interval

According to the different SFG chewing frequencies of the respondents, five SFG chewing frequency levels were defined: "do not use" (0 chewing occasions/week), "infrequent" (0.5 chewing occasions/week), "light" (3.5 chewing occasions/week), “moderate” (7 chewing occasions/week), and “heavy” (14 chewing occasions/week). One-way analysis of variance of chewing frequency and DMFT was carried out with SPSS, version 23, to obtain the mean DMFT corresponding to different chewing frequency levels. STATA SE 14.0 (Stata Corp) was used to fit mean DMFT and annual SFG consumption data by curve estimation, and the distribution relationship between DMFT and annual SFG consumption and related parameters were reported with 95% confidence intervals (CIs) (Fig. 1).

### Dental expenditure in the last 12 months

The questionnaire completed by the participants evaluated dental expenditure in the past year, including the total cost of dental treatment, the cost of prevention, the cost of filling dental caries, the cost of root canal treatment, the cost of crowns and bridges, and the cost of implant restoration. The cost of filling caries was divided by filled teeth (FT = 0.625) to obtain the cost of filling per tooth, which was used to represent dental expenditure per tooth due to caries.

### Average cost savings per person

**Fig. 2:**
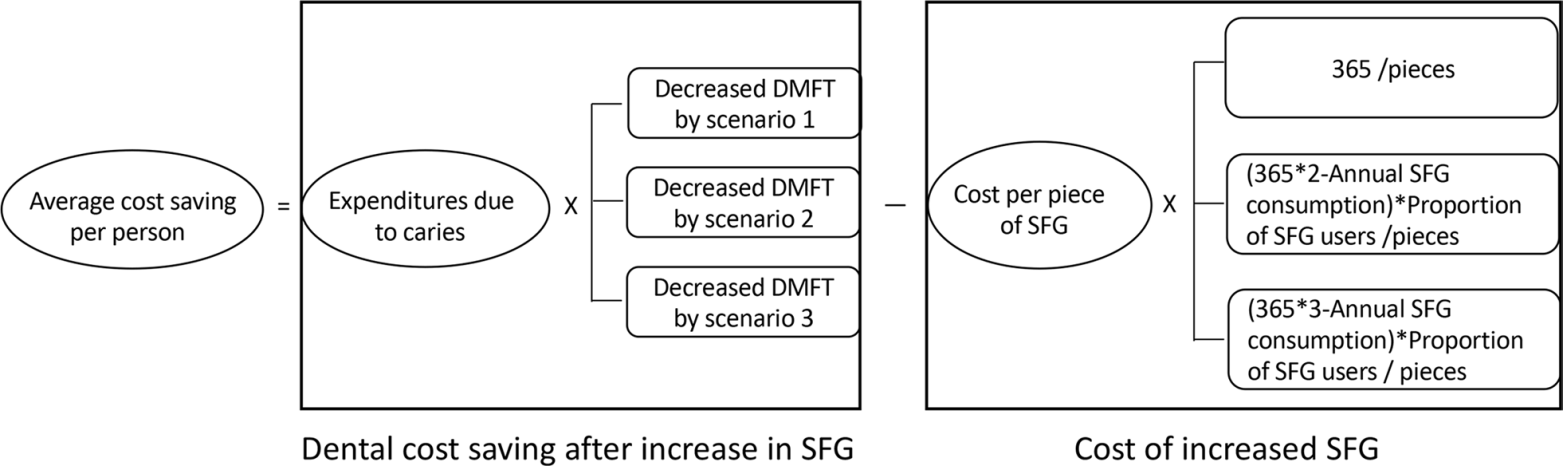
The formula of potential cost savings per person per year by scenario. Abbreviations: SFG, Sugar-Free Gum; DMFT, decayed, missing and filled teeth in permanent dentition

The average cost savings per person were calculated by the dental cost savings after an increase in SFG consumption minus the cost of increased SFG consumption (Fig. 2).

According to the annual consumption of SFG (43,092,965.76 kg), total sales turnover (8,143,236,488.67 RMB) (1,230,579,455.48USD) and the average weight of each piece of SFG (1.45 g) provided by Wrigley company, the price of each piece of SFG (0.27 RMB) (0.04USD) can be calculated. In an attempt to obtain the cost of increased SFG consumption, the price of each piece of SFG was multiplied by the increased per capita consumption of SFG per year (PCC).

Based on the relationship between DMFT and annual SFG consumption, potential dental cost savings after an increase in SFG consumption were estimated.

### Total national cost savings

According to the results of the sixth census in 2010, the total population aged 12 to 15 years and over 18 years in 31 provinces was 1.037 billion [22], which was multiplied by the per capita annual cost savings to obtain the annual total national cost savings.

### Increase in SFG consumption

To explore the impact of the increase in the consumption of SFG, the study designed three different forms for analysis:

The first scenario individually evaluated each person in the model population (apart from the individuals currently not using SFG) using one more piece of SFG per day.

The second scenario was that all members of the model population used two pieces of SFG a day.

The third scenario simulated increased SFG use for the entire model population to three pieces a day.

### Sensitivity analysis

To evaluate the reliability of the results, estimates of important parameters in the model were changed, including the price (0.09–0.39 RMB) (0.01–0.06 USD) for per piece of common brands of SFG in the Chinese market (Additional file 1: Supplementary table); the expenditure due to caries (54–450 RMB) (8.16–68.00 USD); and the coefficients for the dose–response relationship between DMFT and SFG annual consumption.

## Results

Table 1 shows the present situation regarding chewing SFG and corresponding caries prevalence among adolescents aged 12 to 15 years and adults aged 18 years and older in China. According to the results of different SFG chewing frequencies of the respondents, more than half of the survey population do not have the habit of using SFG. The proportion of people who used SFG heavily was only 3.1%. In general, as the chewing frequency of SFG increased, the corresponding population proportion gradually decreased, as did DMFT incidence. Decreased DMFT was observed in the dose–response relationship between DMFT and annual SFG consumption and the relationship conforms to the following exponential equation (Fig. 1):

**Table 1:** Chewing frequency behaviors and caries status in China

Group	Group definition	Annual SFG consumption (pieces)	Proportion of SFG users	Mean DMFT
Group1: No use	0 chewing occasions per week	0	56.50%	2.303
Group2: Infrequent use	0.5 chewing occasions per week	26	20.90%	1.707
Group3: Light use	3.5 chewing occasions per week	182	15.00%	1.677
Group4: Moderate use	7 chewing occasions per week	365	4.50%	1.517
Group5: Heavy use	14 chewing occasions per week	730	3.10%	0.829

*SFG* Sugar-Free Gum, *DMFT* decayed, missing and filled teeth in permanent dentition

$$ \begin{aligned}    & y = 2.172e^{{ - 0.00138x}}  \\     & R^{2}  = 0.6869 \\    & (95\% \; {\text{CI}}\;2.159 \sim 2.185; - 0.001444 \sim - 0.001316) \\  \end{aligned}  $$

Table 2 presents the cost of dental care in different cities over the past 12 months. Nationwide, the expenditure due to caries per tooth was 220 RMB (33.25 USD) (Additional file 2).

**Table 2:** Costs for dental treatment per capita and dental expenditure due to caries per tooth in last 12 months by region

Region	cost for prevention (median)	cost for restoration (median)	cost for RCT (median)	cost for extraction (median)	cost for crown (median)	cost for bridge (median)	cost for implant (median)	dental expenditure (median)	expenditure due to caries (median)
Beijing	¥500 ($75.56)	¥200 ($30.22)	¥600 ($90.67)	¥500 ($75.56)	¥4,500 ($680.03)	¥950 ($143.56)	¥1,000 ($151.12)	¥1,100 ($166.23)	¥50 ($7.56)
Hubei	¥120 ($18.13)	¥290 ($43.82)	¥500 ($75.56)	¥400 ($60.45)	¥2000 ($302.23)	¥0 ($0)	¥17,000 ($2,568.98)	¥500 ($75.56)	¥170 ($25.69)
Guangzhou	¥0 ($0)	¥400 ($60.45)	¥475 ($71.78)	¥300 ($45.34)	¥3,000 ($453.35)	¥0 ($0)	¥0 ($0)	¥1,000 ($151.12)	¥400 ($60.45)
Xinjiang	¥700 ($105.78)	¥725 ($109.56)	¥1000 ($151.12)	¥200 ($30.22)	¥5,900 ($891.59)	¥2,000 ($302.23)	¥110,000 ($16,622.84)	¥818 ($123.61)	¥250 ($37.78)
Average	¥450 ($68.00)	¥500 ($75.56)	¥1,000 ($151.12)	¥400 ($60.45)	¥1,800 ($272.01)	¥1,200 ($181.34)	¥17,000 ($2,568.98)	¥1,000 ($151.12)	¥220 ($33.25)

The cost was converted according to the 2018 Chinese Yuan (RMB) to the USD exchange rate that 100 USD was equivalent to 661.74 RMB (Data resource: China statistical yearbook2019 18–8: http://www.stats.gov.cn/tjsj/ndsj/2019/indexch.htm)

*RCT* root canal treatment

Table 3 displays the potential cost savings under three different scenarios. With the increase in PCC, the average cost savings per person could range from 45.95 RMB (6.94 USD) per year (chewing 2 pieces of SFG per day) to 67.41 RMB (10.19 USD) per year (chewing 3 pieces of SFG per day). Nationally, the 1.037 billion young teenagers aged 12 to 15 years and adults aged 18 years and older currently chewing SFG could save 21.51 billion to 31.55 billion RMB (3.25 billion to 4.77 billion USD) annually (Additional file 3).

**Table 3:** Potential cost saving by scenario (per capita)

	Scenario 1	Scenario 2	Scenario 3
Current PCC	28.67	28.67	28.67
Increased PCC	365.00	245.45	403.34
Costs of increased SFG	¥98.55($14.89)	¥66.27($10.01)	¥108.90($16.46)
Current DMFT	2.00	2.00	2.00
Decreased DMFT	0.74	0.51	0.80
Expenditure due to caries (per tooth)	¥220.00($33.25)	¥220.00($33.25)	¥220.00($33.25)
Dental cost saving after increase in SFG consumption	¥162.01($24.48)	¥112.22($16.96)	¥176.31($26.64)
Average cost saving per person	¥63.46($9.59)	¥45.95($6.94)	¥67.41($10.19)
Total national cost savings (in B RMB/ USD)	¥29.70($4.49)	¥21.51($3.25)	¥31.55($4.77)

*PCC* per capita consumption of sugar-free gum per year, *SFG* Sugar-Free Gum, *DMFT* decayed, missing and filled teeth in permanent dentition

*Scenario 1* The consumption of SFG increased by 1 piece per day among the model population (apart from the individuals currently not using SFG).

*Scenario 2* The consumption of SFG increased to 2 pieces per day among the model population.

*Scenario 3* The consumption of SFG increased to 3 pieces per day among the model population.

The cost was converted according to the 2018 Chinese Yuan (RMB) to the USD exchange rate that 100USD was equivalent to 661.74RMB (Data resource: China statistical yearbook2019 18–8: http://www.stats.gov.cn/tjsj/ndsj/2019/indexch.htm).

Sensitivity analysis showed that when the important parameters mentioned in the Methods were all set at the minimum values, the average annual cost saving per capita was 3.54–4.77 RMB (0.53–0.72 USD) and the total cost savings was 16.59 billion to 22.31 billion RMB (2.51 billion to 3.37 billion USD). (Table 4) When these parameters were the maximum values, the average annual cost savings per capita was 149.40–221.15 RMB (22.58–33.42USD), and the total cost savings nationwide was 699.3 billion to 1035.1 billion RMB (105.68 billion to 156.42 billion USD). (Table 5).

**Table 4:** Sensitivity analysis-potential cost saving by scenario when important parameters were the minimum values

	Scenario 1	Scenario 2	Scenario 3
Current PCC	28.67	28.67	28.67
Increased PCC	365.00	245.45	403.34
Costs of increased SFG	¥32.85($4.96)	¥22.09($3.34)	¥36.30($5.49)
Current DMFT	2.00	2.00	2.00
Decreased DMFT	0.70	0.47	0.76
Expenditure due to caries (per tooth)	¥54.00($8.16)	¥54.00($8.16)	¥54.00($8.16)
Dental cost saving after increase in SFG consumption	¥37.61($5.68)	¥25.63($3.87)	¥41.07($6.21)
Average cost saving per person	¥4.76($0.72)	¥3.54($0.53)	¥4.77($0.72)
Total national cost savings (in B RMB/ USD)	¥22.27($3.37)	¥16.59 ($2.51)	¥22.31($3.37)

Important parameters include the price of each piece of SFG, the expenditure due to caries per tooth and the CI of the parameters in the dose–response relationship between DMFT and SFG annual consumption

*PCC* per capita consumption of sugar-free gum per year, *SFG* Sugar-Free Gum, *DMFT* decayed, missing and filled teeth in permanent dentition

**Table 5:** Sensitivity analysis-potential cost saving by scenario when important parameters were all the maximum values

	Scenario 1	Scenario 2	Scenario 3
Current PCC	28.67	28.67	28.67
Increased PCC	365.00	245.45	403.34
Costs of increased SFG	¥142.35($21.51)	¥95.73($14.47)	¥157.30($23.77)
Current DMFT	2.00	2.00	2.00
Decreased DMFT	0.78	0.54	0.84
Expenditure due to caries (per tooth)	¥450.00($68.00)	¥450.00($68.00)	¥450.00($68.00)
Dental cost saving after increase in SFG consumption	¥348.82($52.71)	¥245.13($37.04)	¥378.45($57.19)
Average cost saving per person	¥206.47($31.20)	¥149.40($22.58)	¥221.15($33.42)
Total national cost savings (in B RMB/ USD)	¥966.39($146.04)	¥699.30($105.68)	¥1,035.12($156.42)

*PCC* per capita consumption of sugar-free gum per year, *SFG* Sugar-Free Gum, *DMFT* decayed, missing and filled teeth in permanent dentition

Important parameters include the price of each piece of SFG, the expenditure due to caries per tooth and the CI of the parameters in the dose–response relationship between DMFT and SFG annual consumption

*Scenario 1* The consumption of SFG increased by 1 piece per day among the model population (apart from the individuals currently not using SFG).

*Scenario 2* The consumption of SFG increased to 2 pieces per day among the model population.

*Scenario 3* The consumption of SFG increased to 3 pieces per day among the model population.

The cost was converted according to the 2018 Chinese Yuan (RMB) to the USD exchange rate that 100USD was equivalent to 661.74RMB (Data resource: China statistical yearbook2019 18–8: http://www.stats.gov.cn/tjsj/ndsj/2019/indexch.htm).

## Discussion

This study is the first to use cross-sectional survey data to analyze the potential economic benefits of SFG in caries prevention. The cross-sectional survey was aimed at providing real world data in China to simulate the dose–response relationship between chewing SFG and DMFT for the economic analysis purpose. Then, the dose–response assumption made by modeling real word data were used to explore the economic benefits of increasing the use of SFG in China. A large number of studies have shown that chewing SFG can prevent the development of dental caries [23–30]. We can therefore assume that an increased SFG consumption could greatly reduce dental care costs due to a potentially reduction of to dental caries. If Chinese adolescents aged 12–15 years and adults aged 18 years and older increased their current frequency of SFG use to 2 pieces per day, it might save 21.5 billion RMB (3.25 billion USD) each year.

The current health economic analysis on the use of SFG to prevent caries is very limited worldwide [18–20]. In a study conducted by Claxton et al. on a 12-year-old British population of 685,000 people, when individuals chewed two to three SFGs a day, the annual cost savings per capita was 1.70–11.97 GBP (2.27–15.96USD) [18]. After increasing the annual per capita consumption of SFG from 111 to 202 in Germany, the annual cost savings per person was approximately 80.82 EUR(95.31USD) [19]. In Reinhard et al.'s study of 25 countries, the overall average annual savings per capita was between 0.21 and 4.74 USD[20] Our research results show that the average annual cost savings per capita was 3.54–221.15 RMB (0.53–33.42 USD), which is basically consistent with the results of previous studies [18–20]. The difference in annual cost savings per capita may be mainly due to differences in the cost of caries treatment in different countries. It can be estimated based on the basic national conditions that China has a total population of approximately 1.4 billion, ranking first in the world. Thus, the potential and effect of cost savings from increasing the use of SFG far exceeds those of other countries.

A number of studies have demonstrated the existence of a dose response relationship, that is, the more gum was chewed, the lower the rates of decay. Two studies on the effect of chewing SFG on the development of dental caries in Chinese residents showed that chewing two to four pieces of gum daily resulted in a reduction in DMFT increment ranging from 35.4% to 47.8% [31, 32]. The reduction in the present analysis is 40% with daily consumption of three pieces of gum. Previous study on SFG economic evaluations globally used existing clinical study results as the relationship assumptions between SFG use and caries reductions. In our study, the relationship between the annual consumption of SFG and DMFT was assumed based on the data we collected from a cross-sectional survey, the survey result was also consistent with those of previous clinical trials conducted in China. Size, taste and type (streaks, pieces) of sugar-free gum were not considered in this study. There is no evidence of a difference between the effects of commonly used sugar substitutes xylitol and sorbitol [33], 34 nor is there any data on the effects of other chewing gum properties.

Our study included only the national population with SFG chewing habits in the model. Therefore, the actual use of SFG in China significantly affects the research results. According to the study of Jing et al. in 2013, the proportion of undergraduates using SFG frequently was 34.4% [35], whereas the proportion of the population with chewing SFG habits regularly in our study was less than 30%. In fact, the frequency of SFG use in China was much lower than that in other Western countries. According to Reinhard et al.'s survey on SFG use in 25 industrialized countries, the annual SFG consumption in China ranks fourth from the bottom, far behind that in Switzerland, Sweden, the United States and other Western countries [20]. Therefore, based on our scenario and population, chewing SFG would be suggested among the public, which may lead to an increase in the rate of SFG use in China and, consequently, a substantial reduction in dental health care expenditures.

In this study, only the cost of restoration was used to replace the cost of caries treatment. There were a mix of teenagers and adults in the study and restorations would be the common denominator as it is unlikely to have crowns, bridges and implants in teenager population although possible in adult population. In fact, the cost of caries-related root canal treatment, crown and bridge restoration, tooth extraction, and dental implant treatment is much higher than the cost of restoration in adult population. Therefore, our results actually underestimate the cost savings and long-term health benefits associated with increased SFG use. Therefore, by increasing SFG use and thus reducing the level of decay development, it is likely that greater long-term savings will be realized than the estimated amounts determined in this analysis.

SFG consumption may vary in different age groups and the process of urbanization. According to previous studies, teenagers and young adults are major consumers of SFG [36]. They might have open and independent consumption attitudes and tending towards consumption ideas of individuation and fashion. Also, they are often willing to try something new. The reason why they buy and chew sugar-free gum may be not necessarily to prevent dental caries, but to freshen their breath. As for urbanization, there are few studies onto it. Base on one study among army men and cadets in China [37], the chewing rate in urban areas was similar to that in rural areas. There are also studies in China suggesting that there were limited resources of SFG in rural areas [37, 38]. It may affect the consumption of SFG in rural areas.

There are some limitations in our study. First, because only the cost of caries restoration was used to represent the treatment cost due to caries and only the potential cost savings that may occur due to the occurrence of dental caries in a relatively short (1 year) period are obtained, the result is likely to underestimate the lifetime possibility cost savings and long-term health benefits. Besides, although the dose–response relationship between SFG annual consumption and DMFT in our study was assumed on the basis of cross-sectional data, we found it is consistent with the results obtained from previous clinical trials conducted in China.

Future research should focus on increasing the sample size to allow the benefits of chewing gum to be distinguished among different age groups, and well-designed clinical trials should be designed to evaluate the effect of SFG on dental caries.

## Conclusion

This study suggests that substantial cost savings could be achieved if SFG use levels were increased in the Chinese population. Though there is no doubt that regular and effective brushing and flossing are still the main measures of dental health, chewing SFG regularly could be considered as an aid to teeth cleaning.

## Supplementary Information

**Additional file 1**. The price for per piece of common brands of SFG in the Chinese market.

**Additional file 2**. Oral health questionnaire for adolescents(12–15 years old).

**Additional file 3**. Oral health questionnaire for adults.

## Data Availability

The datasets used during the current study are available from the corresponding authors on reasonable request.

## Abbreviations

SFG: Sugar-Free Gum
DMFT: Decayed, missing and filled teeth in permanent dentition
WHO: World Health Organization
CI: Confidence interval
