# Supplementary material for: The economic benefits of increased sugar-free chewing gum in China: a budget impact analysis
Source: BMC Oral Health. 2021 Sep 7;21:436. doi: 10.1186/s12903-021-01786-8 (PMC8424996; doi:10.1186/s12903-021-01786-8)
Supplement: Supplementary file 1 — Additional file 1. The price for per piece of common brands of SFG in the Chinese market. [file 12903_2021_1786_MOESM1_ESM.docx]

**Supplementary table**

| Brand | Price of each unit* |
| --- | --- |
| EXTRA Wrigley | ¥0.09-0.22($0.01-0.03) |
| Stride Cadbury | ¥0.17-0.39($0.03-0.06) |
| Xylitol Orion | ¥0.18-0.27($0.03-0.04) |
| Yiya Yake | ¥0.20-0.26($0.03-0.04) |

* Each unit means a piece of SFG in 1.45g.

With the wide range of sales channels and price of SFG, the prices of other brands were all given in a range.

The cost was converted according to the 2018 Chinese Yuan (RMB) to the USD exchange rate that 100USD was equivalent to 661.74RMB（Data resource: China statistical yearbook2019 18-8: <http://www.stats.gov.cn/tjsj/ndsj/2019/indexch.htm>）
