# Supplementary material for: The economic benefits of increased sugar-free chewing gum in China: a budget impact analysis
Source: BMC Oral Health. 2021 Sep 7;21:436. doi: 10.1186/s12903-021-01786-8 (PMC8424996; doi:10.1186/s12903-021-01786-8)
Supplement: Supplementary file 2 — Additional file 2. Oral health questionnaire for adolescents(12–15 years old). [file 12903_2021_1786_MOESM2_ESM.docx]

**Oral health questionnaire (adults)**

Respondent ID No.: □□□□□□□□□□□

School: Grade: Class: Name of respondents:

Survey date: Investigator number: □

**Questions related to oral health behaviors please recall your experiences in the past year.**

**Requirements: Please tick the "√" in the "□".**

1. What is your highest educational background?（**Choose only one answer**）

| 1) □Unschooled | 2) □Primary school |
| --- | --- |
| 3) □Junior middle school | 4) □High school |
| 5) □Secondary specialized school | 6) □Junior college |
| 7) □Undergraduate | 8) □Master or above |

1. How often do you usually eat the following food or drink? (**Choose one answer for each item**)

|  | 6 | 5 | 4 | 3 | 2 | 1 |
| --- | --- | --- | --- | --- | --- | --- |
|  | ≥ twice a day | Once a day | 2 to 6 times a week | Once a week | 1 to 3 times a month | Seldom/never |
| 1) Sugar-free gum | □ | □ | □ | □ | □ | □ |
| 2) Desserts (cookies, cakes, bread) and candy (chocolate, sugary gum) | □ | □ | □ | □ | □ | □ |
| 3) Sweetened beverages (sugar water, carbonated beverages, orange juice, apple juice, lemonade) | □ | □ | □ | □ | □ | □ |
| 4) Sweetened milk, yogurt, milk powder, tea, soy milk, coffee | □ | □ | □ | □ | □ | □ |

1. Do you smoke?（**Choose only one answer**）
   1. 1) □Smoking 2) □Never smoking 3) □Smoking cessation（**If choose item 2 or 3, don't answer questions 4 and 5**）
2. How many years have you been smoking? _______year(s). (**Please fill in an integer, and fill in “N” if you have no idea or refuse to answer**)
3. During the past one month, how many cigarettes did you smoke one day on average?（**Choose only one answer**）

1) □≤1 /day 2) □1-5/day 3) □6-10/day

4) □11-20/day 5) □21-40/day 6) □41/day

1. Do you drink white liquor? (**Choose only one answer**)

1) □Drink every day 2) □drink every week 3) □Drink seldomly

4) □Never drink 5) □have stopped drinking

1. Do you use the following method to clean your teeth? (**Choose one answer for each item**)

|  | 6 | 5 | 4 | 3 | 2 | 1 |
| --- | --- | --- | --- | --- | --- | --- |
|  | ≥ twice a day | Once a day | 2 to 6 times a week | Once a week | 1 to 3 times a month | Seldom/  never |
| 1) Brushing teeth | □ | □ | □ | □ | □ | □ |
| 2) Toothpick | □ | □ | □ | □ | □ | □ |
| 3) Dental floss | □ | □ | □ | □ | □ | □ |

1. Do you use toothpaste when you brush your teeth? (**Choose only one answer**)

1) □Yes 2) □No 3) □Don't know (**If choose item 2 or 3, don't answer question 9**)

1. Do you use fluoride toothpaste when you brush your teeth? (**Choose only one answer**)

1) □Yes 2) □No 3) □Don't know

1. Have you been to the dentist?(**Choose only one answer**)

1) □Yes 2) □No (**If choose item 2, don't answer questions 11-15**)

1. How long has it been since your last visit to the dentist?(**Choose only one answer**)

1) □Within six months 2) □6 to 12 months **(If choose item 1 or 2, don't answer question 16**） 3) □More than 12 months（**If choose item 3, don't answer questions 12-15**）

1. What was the main reason for your last visit to the dentist?(**Choose only one answer**)

1) □Consulting check 2) □Prevention 3) □Treatment 4) □Don't know

1. How much did you spend on dental therapy in the past year? ¥___ yuan（**Please fill in an integer, and fill in “N” if you have no idea or refuse to answer**）

1) Prevention(fluoride, pit and fissure sealant…) ¥_____ 2)Restoration for caries ¥_____

3) Root canal therapy ¥_____ 4) Extraction ¥_____

5) Crown ¥_____ 6) Bridge ¥_____

7) Implant ¥_____

1. What percentage of the above dental expenses did you have to pay on your own? _______% (**Please fill in an integer, and fill in “N” if you have no idea or refuse to answer**)
2. Was the cost of your last dental visit reimbursable?（**Multiple choices**）

| 1) □Basic insurance for urban workers | 2) □Basic medical insurance for non-working  urban residents |
| --- | --- |
| 3) □New Rural Co-operative Medical System | 4) □Commercial insurance |
| 5) □Free medical service | 6) □Reimbursement by other means |
| 7) □Self-paying (No reimbursement) |  |

1. What were the reasons not to have your teeth checked in the past 12 months?（**Multiple choices**）

1) □Nothing wrong with teeth 2) □Dental disease was not serious

3) □Have no time 4) □Economic hardship to pay the dental visits

5) □Dental visits are not reimbursable 6) □There was no dentist nearby

7) □Fear of communicable disease 8) □There was no dentist nearby

9) □Difficult to find a reliable dentist 10) □Difficult to registration

11) □Other reasons

1. Do you have the following medical coverage?**(Choose one answer)**

|  | Yes1 | No2 |
| --- | --- | --- |
| 1)Basic insurance for urban workers | □ | □ |
| 2)Basic medical insurance for non-working urban residents | □ | □ |
| 3)New Rural Co-operative Medical System | □ | □ |
| 4)Commercial insurance | □ | □ |
| 5)Free medical service |  |  |

1. Have you had your teeth cleaned in the past 12 months?

1) □Yes 2) □No**（If choose item 2, don't answer question 19）**

1. What is the pattern of reimbursement of the cost of your teeth cleaning（**Multiple choices**）

1) □Basic insurance for urban workers 2) □Basic medical insurance for non-working urban residents Yes

3) □New Rural Co-operative Medical System 4) □Commercial insurance

5) □Free medical service 6) □Reimbursement by other means

7) □Self-paying (No reimbursement)

1. How much do oral problems affect you in the following ways? **(55-64 age group do not need to answer this question, choose one answer for each question)**

|  | 1 | 2 | 3 | 4 | 5 |
| --- | --- | --- | --- | --- | --- |
| 1) Do you often limit the type and amount of food you eat because of your teeth or dentures? | Very often | Often | Sometimes | Rarely | None |
| 2) Do you have any difficulty biting or chewing your food? |  |  |  |  |  |
| 3) Do you often feel uncomfortable or difficult when you swallow food? |  |  |  |  |  |
| 4) Are your teeth or dentures hindering you speak? |  |  |  |  |  |
| 5) Do you often feel uncomfortable in your mouth when you eat? |  |  |  |  |  |
| 6) Do you often limit your interactions with others due to teeth or dentures? |  |  |  |  |  |
| 7) Do you often feel dissatisfied or unhappy with the appearance of your teeth, gums or dentures? |  |  |  |  |  |
| 8) Do you often take medicine to relieve pain or discomfort in your mouth? |  |  |  |  |  |
| 9) Do you often worry or pay attention to your teeth, gums or dentures? |  |  |  |  |  |
| 10) Do you often feel nervous or uncomfortable in front of others because of your teeth, gums or dentures? |  |  |  |  |  |
| 11) Do you often feel uncomfortable when eating in front of others because of your teeth or dentures? |  |  |  |  |  |
| 12) Are your teeth or gums sensitive to cold, hot or sweet stimuli? |  |  |  |  |  |

1. How do you evaluate your overall health? (**Choose only one answer**)

1) □Excellent 2) □Good 3) □General 4) □Poor

5) □Bad

1. What do you think of your oral and dental health? (**Choose only one answer**)

1) □Excellent 2) □Good 3) □General 4) □Poor

5) □Bad

1. What is your opinion about the following statement? (**choose one answer for each question**)

|  | 1 | 2 | 8 | 9 |
| --- | --- | --- | --- | --- |
|  | Agree | Don't agree | Indifferent | Don't know |
| 1) Oral health is very important to your life | □ | □ | □ | □ |
| 2) Regular oral examinations are necessary | □ | □ | □ | □ |
| 3) Good or bad teeth are innate and have little to do with their own protection | □ | □ | □ | □ |
| 4) The prevention of dental disease mainly depends on the students themselves | □ | □ | □ | □ |

1. Do you think the following statement is true? (**choose one answer for each question**)

|  | 1 | 2 | 8 |
| --- | --- | --- | --- |
|  | Correct | Not correct | Don't know |
| 1) Bleeding gums is normal when brushing teeth | □ | □ | □ |
| 2) Bacteria can cause gingivitis | □ | □ | □ |
| 3) Brushing teeth has no effect on preventing gingivitis | □ | □ | □ |
| 4) Bacteria can cause tooth decay | □ | □ | □ |
| 5) Eating sugar can cause tooth decay | □ | □ | □ |
| 6) Fluoride has no effect on the protection of teeth | □ | □ | □ |
| 7) The pit and groove seal can protect the teeth | □ | □ | □ |
| 8) Oral diseases may affect overall health | □ | □ | □ |
| 9) Chewing SFG could offer protection against dental caries | □ | □ | □ |
| 10) Chewing SFG is harmful to teeth | □ | □ | □ |

1. Have you ever been diagnosed with the following chronic diseases by the doctor?（**Multiple choices**）

1)stroke 2)Diabetes 3)Hypertension

4)Heart disease 5) Chronic obstructive pulmonary disease 6)Other, please specify____

7)None 8)Don’t know

1. How many people are there in your family together?________( **Please fill in an integer, and fill in “N” if you have no idea or refuse to answer**).
2. The total income of your family in the past 12 months? ______ Ten thousand yuan/year(**Please fill in an integer, and fill in “N” if you have no idea or refuse to answer**).

**Thank you very much for your cooperation！**
