# Supplementary material for: The economic benefits of increased sugar-free chewing gum in China: a budget impact analysis
Source: BMC Oral Health. 2021 Sep 7;21:436. doi: 10.1186/s12903-021-01786-8 (PMC8424996; doi:10.1186/s12903-021-01786-8)
Supplement: Supplementary file 3 — Additional file 3. Oral health questionnaire for adults. [file 12903_2021_1786_MOESM3_ESM.docx]

**Oral health questionnaire (adolescents)**

Respondent ID No.: 

School: Grade: Class: Name of respondents:

Survey date: Investigator number: □

The classmates:

Hello, everyone. In order to further improve the oral health care for children and teenagers, we would like to know your thoughts and practices on oral health care. This survey has nothing to do with your academic performance, and the survey results will not be shared with parents and teachers. **Questions related to oral health behaviors please recall your experiences in the past year.** I hope you will answer truthfully as the question asks. Thank you.

**Requirements: Please tick the "√" in the "□".**

1. Are you an only child? (**Choose only one answer**)

1) □Yes 2) □No

1. What is your father's highest educational qualification? (**Choose only one answer**)

| 1) □Unschooled | 2) □Primary school | 3) □Junior middle school | 4) □High school |
| --- | --- | --- | --- |
| 5) □Secondary specialized school | 6) □Junior college | 7) □Undergraduate | 8) □Master or above |
| 9) □No father or don't know | |  |  |

1. What is your mother's highest educational qualification? (**Choose only one answer**)

| 1) □Unschooled | 2) □Primary school | 3) □Junior middle school | 4) □High school |
| --- | --- | --- | --- |
| 5) □Secondary specialized school | 6) □Junior college | 7) □Undergraduate | 8) □Master or above |
| 9) □No mother or don't know | |  |  |

1. Do you brush your teeth? (**Choose only one answer**)

1) □Yes 2) □Brush teeth occasionally or never (**those who chose this answer need not answer questions 5-7**)

1. How often do you brush your teeth? (**Choose only one answer**)

1) □≥Twice a day 2) □Once a day 3) □Not every day

1. Do you use toothpaste when you brush your teeth? (**Choose only one answer**)

1) □Yes 2) □No 3) □Don't know (**those who chose “No or don't know” need not answer questions 7**)

1. Do you use fluoride toothpaste when you brush your teeth? (**Choose only one answer**)

1) □Yes 2) □No 3) □Don't know

1. Do you use dental floss? (**Choose only one answer**)

1) □Never 2) □Occasionally 3) □Every week 4) □Every day

1. How often do you usually eat the following food or drink? (**choose one answer for each question**)

|  | 6 | 5 | 4 | 3 | 2 | 1 |
| --- | --- | --- | --- | --- | --- | --- |
|  | ≥ twice a day | Once a day | 2 to 6 times a week | Once a week | 1 to 3 times a month | Seldom/never |
| 1) Sugar-free gum | □ | □ | □ | □ | □ | □ |
| 2) Desserts (cookies, cakes, bread) and candy (chocolate, sugary gum) | □ | □ | □ | □ | □ | □ |
| 3) Sweetened beverages (sugar water, carbonated beverages, orange juice, apple juice, lemonade) | □ | □ | □ | □ | □ | □ |
| 4) Sweetened milk, yogurt, milk powder, tea, soy milk, coffee | □ | □ | □ | □ | □ | □ |

1. Do you smoke? (**Choose only one answer**)

1) □Every day 2) □Every week 3) □Occasionally 4) □Never

1. How do you evaluate your overall health? (**Choose only one answer**)

1) □Excellent 2) □Good 3) □General 4) □Poor

5) □Bad

1. What do you think of your oral and dental health? (**Choose only one answer**)

1) □Excellent 2) □Good 3) □General 4) □Poor

5) □Bad

1. Have you ever broken your tooth? (**Choose only one answer**)

1) □Yes 2) □No 3) □Don't know (**those who chose “No or don't know” need not answer questions 14**)

1. Where did your tooth get hurt?（**Multiple choices**）

1) □On campus 2) □Outside the campus

1. Have you had a toothache in the past 12 months? (**Choose only one answer**)

1) □Often 2) □Occasionally 3) □Never 4) □Can't remember

1. Have you been to the dentist?(**Choose only one answer**)

1) □Yes 2) □No (**Those who choose the answer need not answer questions 17 or 18**)

1. How long has it been since your last visit to the dentist?(**Choose only one answer**)

1) □Within six months 2) □6 to 12 months

3) □More than 12 months(**Those who choose the answer need not answer questions 18**)

1. What was the main reason for your last visit to the dentist? (**Choose only one answer**)

1) □Consulting check 2) □Prevention 3) □Treatment 4) □Don't know

1. Do you think the following statement is true? (**choose one answer for each question**)

|  | 1 | 2 | 8 |
| --- | --- | --- | --- |
|  | Correct | Not correct | Don't know |
| 1) Bleeding gums is normal when brushing teeth | □ | □ | □ |
| 2) Bacteria can cause gingivitis | □ | □ | □ |
| 3) Brushing teeth has no effect on preventing gingivitis | □ | □ | □ |
| 4) Bacteria can cause tooth decay | □ | □ | □ |
| 5) Eating sugar can cause tooth decay | □ | □ | □ |
| 6) Fluoride has no effect on the protection of teeth | □ | □ | □ |
| 7) The pit and fissure seal can protect the teeth | □ | □ | □ |
| 8) Oral diseases may affect overall health | □ | □ | □ |
| 9) Chewing SFG could offer protection against dental caries | □ | □ | □ |
| 10) Chewing SFG is harmful to teeth | □ | □ | □ |

1. What is your opinion about the following statement? (**choose one answer for each question**)

|  | 1 | 2 | 8 | 9 |
| --- | --- | --- | --- | --- |
|  | Agree | Don't agree | Indifferent | Don't know |
| 1) Oral health is very important to your life | □ | □ | □ | □ |
| 2) Regular oral examinations are necessary | □ | □ | □ | □ |
| 3) Good or bad teeth are innate and have little to do with their own protection | □ | □ | □ | □ |
| 4) The prevention of dental disease mainly depends on the students themselves | □ | □ | □ | □ |

1. How much did your oral problems affect you in the following areas in the past 6 months? (**choose one answer for each question**)

|  | 1 | 2 | 3 | 4 | 5 |
| --- | --- | --- | --- | --- | --- |
|  | serious effect | General effect | Slight effect | No effect | Don't know |
| 1) Eat | □ | □ | □ | □ | □ |
| 2) Pronunciation | □ | □ | □ | □ | □ |
| 3) Brush your teeth or rinse your mouth | □ | □ | □ | □ | □ |
| 4) Do the housework | □ | □ | □ | □ | □ |
| 5) Go to school | □ | □ | □ | □ | □ |
| 6) Sleep | □ | □ | □ | □ | □ |
| 7) A toothy smile | □ | □ | □ | □ | □ |
| 8) Easily upset | □ | □ | □ | □ | □ |
| 9) Interpersonal communication | □ | □ | □ | □ | □ |

1. How many oral health classes did you take in school last semester? _____times. (**please fill in an integer, do not know or refuse to answer please write "N"**)
2. How many classed have you missed due to dental visits in the last year? _____class(es). (**please fill in an integer, do not know or refuse to answer please write "N"**)

**Thank you very much for your cooperation！**
